# The good, the bad and the ugly faces of cyanobiphenyl mesogens in selected tracks of fundamental and applied liquid crystal research

**DOI:** 10.1080/02678292.2023.2292621

**Published:** 2023-12-19

**Authors:** Jan P. F. Lagerwall

**Affiliations:** Experimental Soft Matter Physics group, Department of Physics and Materials Science, University of Luxembourg, Luxembourg, Luxembourg

**Keywords:** Nematic liquid crystal, cyanobiphenyl, 5CB, confinement effects on liquid crystals, liquid crystal-based sensors, liquid crystal-nanoparticle composites

## Abstract

Liquid crystal-forming cyanobiphenyls are truly extraordinary molecules that have had an enormous impact on liquid crystal research and applications since they were first synthesised. This impact is, on the one hand, due to the exceptionally convenient physical properties of the main characters, 5CB and 8CB, allowing easy experiments at room temperature, as well as their commercial availability at reasonable cost. On the other hand, the cyanobiphenyl chemical structure leads to some quite peculiar characteristics in terms of organisation at the molecular scale, which are sometimes well recognised and even utilised, but often the awareness of these peculiarities is not strong. This perspective article reviews the use of cyanobiphenyls in making liquid crystal shells and liquid crystal core fibres, in sensing, as a medium for simultaneously aligning and dispersing carbon nanotubes, and as highly useful solvents for reactive mesogens that can be polymerised into anisotropic networks. This choice is very much motivated by how cyanobiphenyls have impacted our group’s research throughout the years, which is the basis for the examples I provide. Nevertheless, I believe they serve well to illustrate the immense usefulness of cyanobiphenyls in innovating research and applications related to liquid crystals.

## 1. Introduction

I believe it was Alberto Fernandez-Nieves who in a talk at the 2011 APS meeting described 5CB as ‘the fruit fly of liquid crystal research’. This description stuck with me, as 5CB has really become *the* liquid crystalline material for many researchers. In particular, this holds for physicists and engineers who may not be so interested in analysing what is happening at the molecular scale but treat liquid crystallinity as a continuum concept where they only need to have *a* liquid crystal (LC), no matter which one as long as it is convenient to work with. And 5CB truly lives up to the last criterion: a single-component material, thus with sharp transition temperatures and none of the phase coexistence phenomena that you always encounter with mixtures, developing a nematic phase practically at room temperature, with low viscosity, low volatility and low toxicity, and nowadays at reasonably low cost. For the slightly more adventurous researchers who wish to go beyond the nematic phase and also study smectic order, the corresponding fruit fly is the second most common *n*CB homologue, 8CB, exhibiting a SmA phase conveniently near room temperature.

**Figure 1.**
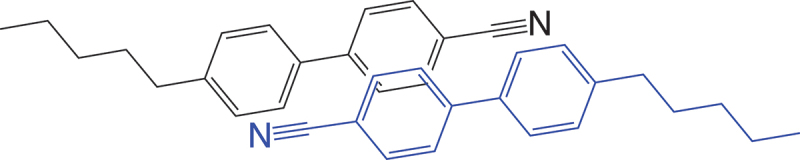
(Colour online) Cartoon illustrating how 5CB molecules spontaneously dimerize in an antiparallel fashion to maximize the overlap of aromatic regions. The dimer fulfills the classic Vorländer paradigm for mesogenic design, with a stiff core surrounded by flexible end chains on both sides.

Nevertheless, just like a fruit fly is not a good model for all kinds of biological life, 5CB has its limitations in representing nematic LCs in a generic sense, because it exhibits some very peculiar features that are intimately linked to its molecular design. For instance, most users of 5CB consider it a room temperature nematic, often not reflecting over the contradiction that their room temperature may be about 20 ∘C, yet the melting point of 5CB is 23 ∘C; the nematic phase of 5CB has an incredible tendency to supercool. Moreover, ask a liquid crystal scientist what are the characteristics of mesogens, i.e. liquid crystal-forming molecules, and you will very likely hear a rule that dates back to Vorländer [1,] namely that they should generally have rigid rod-shaped core and flexible end chains. Note the plural ‘s’ in *chains*: the expectation is that the molecule is qualitatively symmetric, with a linear core, typically aromatic, and an aliphatic chain sticking out from each end. Then ask them to show an example of such a molecule, and they will very likely show 5CB, which is highly asymmetric with *one* aliphatic end chain, the other end being an aromatic and inflexible cyano group. This means that 5CB is a rather poor pedagogical example in the sense that it causes a logical mismatch between the Vorländer archetypal mesogen and the structure that a student is shown to exemplify. More importantly, the asymmetric molecule design has enormous implications for the way 5CB, and any of the many *n*CB and *n*OCB homologues organise in an LC phase: They dimerise with antiparallel order [2,3], see Figure 1, thus living up to the Vorländer paradigm as *couples*, not as individual molecules. This is one of the many ways in which *n*(O)CBs are very special mesogens, with many beneficial aspects but sometimes with quite particular problems, and which in any case renders them questionable as ‘archetypal’ mesogen, despite 5CB being the go-to choice as the standard LC in so many situations.

I have written this perspective article very much as a personal account of how 5CB, 8CB and other *n*(O)CBs as well as *n*(O)CB-based mixtures have impacted the research of my own group, our collaborators, and the communities that we have been active in, throughout the years. Sometimes the impact has been positive, as when 5CB or 8CB has enabled the success of pilot experiments, the observation of fascinating phenomena, or the development of useful applications. Sometimes it was less helpful, in particular when we attempted to translate these phenomena and applications to non-*n*(O)CB liquid crystals and we realised the hard way that the earlier results relied on the peculiar characteristics of *n*(O)CBs; with other mesogens we could not reproduce the results. I have selected five themes in which *n*(O)CBs have largely dominated the research, in our group and elsewhere, most often without much thought of whether this has created a biased view or not. Sometimes experiments with other mesogens have shown that this is the case, sometimes the situation with alternative mesogens remains to be systematically investigated.

## 2. Liquid crystal shells made of *n*CB

**Figure 2.**
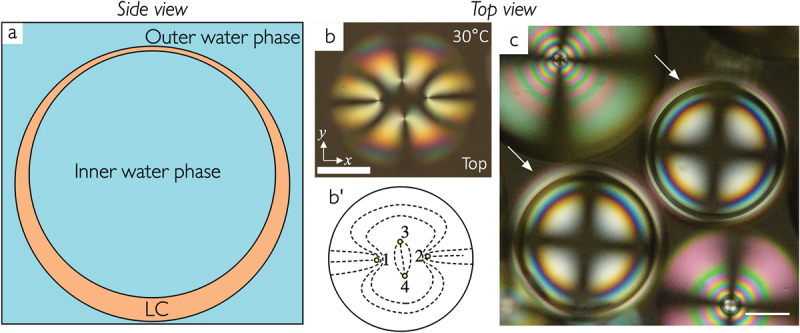
(Colour online) (a) schematic of a liquid crystal shell with water-based surrounding isotropic phases, drawn from the side to illustrate the buoyancy-induced asymmetry that renders the shell thickest at the bottom and thinnest at the top, or vice versa. (b) a fully tangential-aligned nematic shell near room temperature of E7 stabilized by a water solution of the amphiphilic polymer F127 on both sides, viewed from the top between crossed polarizers. The four topological defects are collected near the thinnest point (the top), as illustrated in the director field sketch in (b ′). Scale bar: 50 *μ*m. Reproduced from [4] on CC by 4.0 license. (c) nematic droplets and shells (highlighted with arrows) formed by the fully aliphatic mesogen 1-methoxy-4-(4-pentylcyclohexyl) cyclohexane stabilized by a water solution of PVA. The very different texture compared to (b) shows that this LC aligns with normal boundary conditions to water, in contrast to *n*CB-based LCs. Scale bar: 100 *μ*m. Reproduced from [5] on CC by 3.0 license.

Liquid crystal shells are self-closing spheres of liquid crystals that contain and are surrounded by immiscible isotropic liquids, often an aqueous solution of a suitable interface stabiliser like polyvinyl alcohol (PVA) or a surfactant, see Figure 2. They are fascinating from a whole range of perspectives [6–10], but the aspect that initially sparked the interest is the fact that tangentially aligned nematic shells, according to the Poincaré-Hopf theorem, must develop topological defects whose total strength amounts to +2, at both interfaces, inside and outside. There are several defect configurations that can fulfil this, the most intriguing one perhaps being that of four +1/2 disclinations arranged tetrahedrally to minimise the elastic distortion energy in the shell. Nelson conceived the idea that the defects might serve as anchors for ligand molecules that are able to bond to each other, and the expected tetrahedral configuration of the defects would then turn such a functionalised nematic sphere into a reactive particle that could serve as building block for colloidal crystals with diamond-like lattice [11], an attractive but elusive structure in the realm of photonic crystals.

The first practical realisation of liquid crystal shells was reported by the group of David Weitz in 2007 [12]. It then turned out that the targeted tetrahedral defect configuration is much more difficult to achieve in practice, as the freedom of the inner isotropic liquid droplet to move away from the centre of the shell makes the latter asymmetric, with a thinnest and a thickest point, and typically all defects in a shell with tangential alignment on both sides are collected near the thinnest point (Figure 2(a–b)). Only by making the shell very thin can the tetrahedral configuration be stabilised [13], and the size is generally orders of magnitude too large to be of relevance for colloidal crystals. Lopez-Leon and co-workers recently succeeded in making exceptionally small liquid crystal shells, reaching a diameter on the order of 10 *μ*m [14], but the targeted range would still require a factor 10 smaller. However, even if their application as a basis for a new colloidal chemistry may not be realistic, LC shells provide ample application opportunities of different nature, not least when using cholesteric LCs [7,8].

To form liquid crystal shells, one generally flows the liquid crystal material as an intermediate phase between two immiscible isotropic liquids in a nested capillary device, the flow rates being reasonably fast, on the order of mL/hr. Because it can take some time before the three-phase flow has been properly set up and stabilised, comparatively large volumes are needed for each experiment, hence one needs a liquid crystal that is available in large quantities at acceptable cost. Moreover, since temperature control of the entire microfluidic setup is challenging, in particular the tubing between reservoir and device, with its large surface-to-volume ratio, the material should be liquid crystalline and have a rather low viscosity at room temperature. Considering these criteria, 5CB comes across as an ideal compound for making nematic liquid crystal shells, and indeed this was the compound used for the first experiments by Alberto Fernandez-Nieves et al. [12], as well as for many follow-up papers. I had the fortune to attend David Weitz's talk about liquid crystal shells at the 2007 International Soft Matter Conference in Aachen, and this inspired me to start working in this field also in my group. In order to break new ground, we decided to explore smectic LCs, with which I had been working throughout my PhD and post-doc years, and so an obvious choice was … 8CB. Indeed, our first two papers on LC shells used 8CB as mesogen [15,16]. Unbeknownst to us, Lopez-Leon and Fernandez-Nieves had taken this step at the same time, and they submitted their first study on 8CB shells only a month after us, and eventually our two papers were published back-to-back in the same issue of Physical Review Letters [16,17].

My initial aim in the work on smectic shells was, however, to explore how topological defects in a radially aligned SmC phase would behave, as here the defects would not be in the primary director but in the so-called c-director, indicating the tilt direction of the director with respect to the SmC layer normal. This is an example where *n*(O)CBs reach their limit: no *n*(O)CB mesogen, or mixture thereof, develops a SmC phase. We had to explore alternative chemistries and found a quite convenient system in a binary mixture of phenylpyrimidine-based mesogens. With these, we could prepare shells stabilised by ordinary ionic surfactants and we published the first study of SmC shells in 2013 [18]. The study was not very conclusive; however, a reason being that the phenylpyrimidine mixture was not nearly as easy to work with in shell geometry as 8CB. Although we later succeeded in making also tangentially aligned SmC shells from similar mixtures (manuscript soon to be submitted), the stability of the phenylpyrimidine shells was even lower in this configuration. This was the first indication that *n*(O)CBs may not just be convenient due to their temperature ranges, low viscosity and availability in large volumes at reasonable cost, but they also form much more stable shells than many other mesogens.

We encountered the next strong hint that there is something very special about *n*(O)CBs, making their moulding into shells particularly easy, when we tried to make nematic liquid crystal elastomer (LCE) shells with controlled topological defect configuration using the side-on side-chain reactive mesogen that was first introduced by Patrick Keller’s group [19]. Together with Rudolf Zentel’s team, we had previously succeeded in making LCE shells based on this mesogen [20], but the control of the topological defects was limited since they were photopolymerised very rapidly during flow. We now want to produce the shells without polymerising them immediately, instead leaving them to anneal in the monomeric state until they adapt to the equilibrium topological defect configuration dictated by the choice of stabilisers and the shell geometry [13], but it turned out that all shells were breaking directly after production. Given that we at this stage had years of experience with *n*(O)CB-based shells produced under very similar conditions without having any issues with shell stability, we thought to mix in a small amount of an *n*(O)CB mesogen with similar phase sequence to the LCE precursor mixture to see if this improved the shell stability. The results were spectacular: by adding only 1 mol-% of 7OCB, the problems were gone! My students working in the lab, Anjali Sharma and Andy Stoffel, could now easily produce large quantities of LCE precursor shells which survived for days of annealing until equilibrium defect configurations were reached, and then they could photopolymerise them into intact LCE shells [21]. Apparently the amphiphilic nature of 7OCB (most likely any other *n*OCB or *n*CB would have had the same stabilising effect) led it to predominantly populate the interfaces to water, thereby greatly adding to the stability of the shells.

These results prompted us to do a systematic investigation of several non-*n*(O)CB mesogens and mixtures in LC shells [5], allowing us to confirm that, indeed, the choice to start making LC shells using 5CB was a very fortunate serendipitous choice. In the new study, we focused on shells stabilised by ∼85%-hydrolysed polyvinyl alcohol (PVA), dissolved into water at a concentration of 1 mass-%, because this is the standard choice for making tangential-aligned LC shells, as required to see topological defects developing in a nematic phase. While essentially any LC can be stabilised into droplets or shells using appropriately chosen surfactants, they adsorb radially at the water – LC interface, imposing normal alignment of the director. For this reason, we could not use surfactants, and PVA was then the natural choice.

It turned out that the non-*n*(O)CB-based LCs investigated in this study yield shells with dramatically reduced stability (rupture within minutes to hours) when surrounded by aqueous PVA solutions. However, the impact of chemical structure on the experiment even extends beyond the aspect of shell stability: while the conventional wisdom has long been that LCs adopt tangential alignment in contact with water^1^ we found that a nematic LC formed by entirely non-aromatic mesogens aligns normally to the LC – water interface (Figure 2(c)), in shells as well as droplets! This was truly surprising, to us and others, but then the classic experiments to test how an LC aligns with respect to water were conducted with – you probably guessed it—5CB [22].

The story with LC shells teaches us that 5CB and other members of the *n*(O)CB family are extremely useful, facilitating experiments by their physical as well as chemical properties, but one must be careful about drawing conclusions about how ‘liquid crystals behave’ from experiments conducted solely on *n*(O)CBs. Remove the cyano group and much will change and remove all aromatic groups and an entirely different behaviour may arise, although the phase is still a nematic liquid crystal.

## 3. Confining *n*CB into tubes

**Figure 3.**
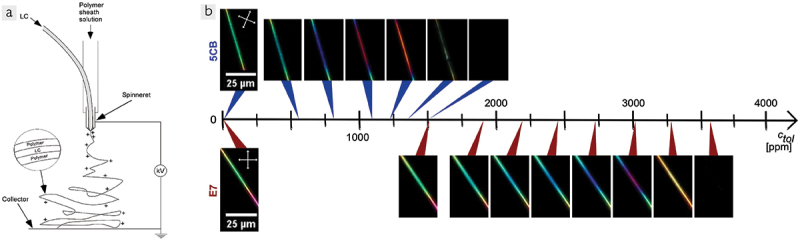
(Colour online) (a) schematic illustration of the coaxial electrospinning process for making LC-filled polymer fibers. Reproduced from [23] on CC BY 2.0 license. (b) polarizing microscopy images of electrospun fibers with PVP sheath and cores of 5CB (top) and E7 (bottom), in the absence of any VOC (left) and upon increasing exposure levels of toluene vapor. As the concentration of toluene increases, the birefringence of the LC core decreases, causing a color change on the way to inducing a transition to isotropic phase (dark image). The required exposure levels are much greater for E7 than for 5CB core, reflecting the clearing temperature far above room temperature of the former, while the latter clears only a few Kelvin over the operation temperature. Reproduced from [24] on CC BY 4.0 license.

In 2006, I had the pleasure of listening to a conference talk by Jesse McCann, PhD candidate of Prof. Younan Xia at the University of Seattle (at the time), describing how they made composite cylindrical fibres with a short-chain alkane core surrounded by a polyvinylpyrrolidone (PVP) solid polymeric sheath using coaxial electrospinning. I was deeply fascinated by this work, not least due to the beautiful physics that governs the spinning process (Figure 3(a)), with electrostatic self repulsion leading to a composite jet of alkane surrounded by PVP solution being ejected from a dual-liquid droplet and moving towards a grounded collector, stretching enormously in the process, all while the Rayleigh – Plateau instability must be prevented from breaking up the jet at the inner as well as the outer interface before enough solvent has evaporated from the sheath solution for it to solidify and thus make the cylindrical core – sheath structure permanent. But equally fascinating were the application-related opportunities arising from this unconventional way of encapsulating a functional liquid; in McCann’s work they utilised the solid – liquid transition of the alkane to make electrospun fibre mats that function as highly efficient heat insulators, taking advantage of the latent heat released or absorbed when the core changes phase. I immediately wondered if we can use this for making liquid crystal devices with a very unusual form factor and thus asked Mr. McCann after his talk if there might be interest in exploring this together. Fortunately, both he and Prof. Xia were positive about this, and a few months later I flew to Seattle to do the first ever experiments with liquid crystal core – polymer sheath coaxial electrospinning in their labs.

The first critical question arising when preparing for this experiment was obviously which liquid crystal to use. As with shells, with electrospinning you need large amounts of liquids, because again this is a continuous flow process and it can take quite some time before the liquid flow rates and voltages are all properly adjusted, and the spinneret may clog up in the process, requiring cleaning. In this case I decided to use a commercial multicomponent mixture, RO-TN-403/015S, exhibiting a broad temperature range nematic phase, extending well below and well above room temperature, that had been developed by Roche for use in twisted nematic displays. To this day, I have not been able to find its exact composition, but in a paper by Gerber [25] it is stated that it contains alkyl and alkoxy cyano biphenyls, alkyl cyano terphenyls, cyano phenyl pyrimidines and terpyrimidines, thus it is formulated with *n*(O)CBs and closely related mesogens. My group has used this and similar mixtures in many of our papers, and I was often asked why we were working with these old mixtures that have not been on sale for decades. The reason is pragmatic: I inherited several decilitres of these mixtures from Prof. Gerd Heppke when he retired from TU-Berlin, and therefore these mixtures were extremely convenient as first test LCs when doing experiments where large volumes of a room temperature nematic phase were required. As will become clear below, I was quite fortunate *not* to have chosen 5CB for the first experiments with coaxial electrospinning, although I of course did not know this at the time; it was again a case of pure serendipity to work with the right LC for the first tests.

The first experiments performed with the Xia group served as a proof of principle, demonstrating that LC-filled polymer fibres could be produced by coaxial electrospinning, and we also noticed some interesting effects due to the tight confinement of the LC inside the polymer, such as a significantly expanded temperature range of the nematic order [26]. Over the next few years electrospinning became a major activity in my group, led by my first PhD student Eva Enz who found interesting confinement effects also with cholesterics [27,28] and smectics [23]. With the latter work, we took the step to spin fibres with regular single-*n*CB LCs in the core, in this case 8CB. The first to electrospin 5CB core polymer fibres were Ebru Buyuktanir, John West and Margaret Frey, who did not use a coaxial spinning set-up but instead relied on in-situ phase separation for obtaining the core-sheath structure [29]. Eventually, our group also spun fibres with 5CB core, finding that the resulting composite fibres are highly interesting for sensing of volatile organic compounds (VOCs) such as toluene [30]. With nematic core, the fibre mat scatters light strongly but as the 5CB core goes isotropic upon exposure to toluene – which easily reaches the 5CB throughout the fibre mat due to the exceptionally large surface-to-volume ratio of the electrospun fibre form factor – the scattering is greatly diminished.

The application opportunity in VOC sensing (which I will discuss in more general terms in the following section) motivated us to continue spinning 5CB-filled fibres and study their particularly fast response to VOC exposure seen between crossed polarisers, at macro- and microscopic scale [31], and then also to systematically vary the VOC concentration [32]. Also, the team of West and Jakli explored VOC sensing with their 5CB-filled fibres produced via phase separation [33]. More recently, we showed that the clearing point of 5CB near room temperature is very beneficial for the fast and sensitive responses to VOC exposure, since even a relatively small concentration of VOC contaminant in 5CB pushes the clearing temperature below room temperature, explaining the response [24], see Figure 3(b). Reference experiments with fibres filled with the 5CB-based four-component mixture E7, with clearing temperature around 63°C, confirmed that a much greater toluene concentration is needed to see a response when the clearing temperature is high above the operating temperature.

**Figure 4.**
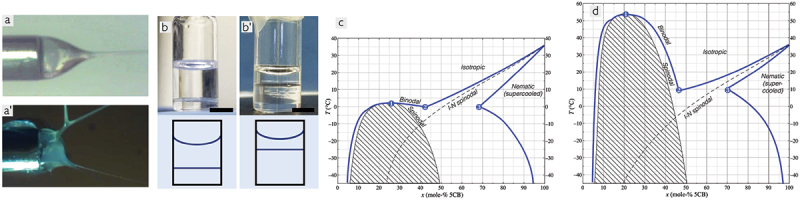
(Colour online) (a) electrospinning at 65% relative humidity, using just an ethanolic PVP solution (top) and a coaxial combination of the same PVP solution as sheath and 5CB as core (bottom). In the latter case, as ethanol mixes with 5CB and water adsorbs from the air, the mixture undergoes strong and complex phase separation, causing catastrophic spinning failure. Reproduced from [34] on CC BY NC ND 4.0 license. (b) macroscopic views of isotropic-isotropic phase separation of 5CB in ethanol with 3 vol.% water added, at 5CB contents 9.6 mole-% (b) and 21 mole-% (b ′), respectively. The phase diagrams without (c) and with (d) 3 vol.-% water are sketched with the same temperature and composition scalings, clearly revealing how a small amount of water raises the phase separation range to high temperatures. Panels (b–d) are reproduced from [35] on CC BY 3.0 license.

But the electrospinning of 5CB-filled fibres was also fraught with problems, causing great frustration for my team members working on this project, in particular Catherine Reyes who patiently investigated the issue deeper. While some days the experiments worked wonderfully, other days no fibres were produced at all because already at the spinneret the LC and the polymer sheath solution did not play well together, see Figure 4(a). It is well known that atmospheric humidity has a great impact on electrospinning, but this on its own could not explain the problems, because we confirmed over and over again that the humidity was far from high enough to cause any problems when pure PVP fibres were spun. It was not until Catherine discovered that the phase diagram of 5CB and ethanol – which was the solvent used to dissolve the PVP, thereby mixing to some extent with 5CB during spinning – exhibits a huge miscibility gap (Figure 4(b–d)) and that the plait point of the spinodal is dramatically raised from about 0°C to more than 50°C if as little as 3 vol.% of water is added [35], that we understood the cause of the problem. Importantly, the phase separation is not just between nematic and isotropic, but for a large composition range the phase separation is between two *isotropic* phases with very different chemical compositions (Figure 4(b)).

Since the coaxial electrospinning process brings the 5CB in contact with the ethanolic PVP solution already in the compound drop from which the fibres are ejected, and since the cooling of the drop due to ethanol evaporation promotes water condensation at the drop – air interface, these conditions are easily fulfilled even under moderately humid atmospheres, yielding dramatic phase separation within the drop during spinning. This leads to strong concentration variations in the PVP solution, which in turn triggers the catastrophic failure of the spinning procedure we encountered so frequently [34]. In other words, although the 5CB is spun as a core liquid and is never directly exposed to the humid atmosphere, its presence disrupts the electrospinning process thanks to its complex phase diagram when mixed with a slightly aqueous ethanol mixture. Similar to 5CB, E7 as core can give rise to problems at elevated humidity, albeit not as severe, while spinning with the RO-TN-403/015S mixture as the core is much less sensitive to air humidity. As the humidity during our first spinning experiments in Seattle could be quite high, it was thus a very fortunate choice that those experiments were not done with 5CB.

Interestingly, it turns out that partial miscibility with a miscibility gap between LC and sheath solution is actually beneficial for coaxial spinning, as it produces a well-defined core – sheath interface with minimum interfacial tension, but the gap should be relatively small and it should be directly between nematic and isotropic phases, not between two isotropic phases of different compositions, as for 5CB and ethanol [36]. We found another Roche mixture, RO-TN-651 (unfortunately, we have not been able to find data on its composition, but it is likely that this also contains a significant fraction of *n*(O)CB mesogens), to have exactly this behaviour in mixtures with ethanol around room temperature and thus conducted a systematic study with this LC mixture in contact with various polymer sheath solutions [36]. While PVP is a convenient polymer from a spinning perspective, its poor mechanical properties and hygroscopicity make it nearly useless for most applications, hence other polymers are needed for making composites for practical use, often requiring solvents other than ethanol, from water to non-polar organic solvents. In this later study, we showed that the relatively long dwelling time with large contact area between core and sheath liquids within the droplet from which spinning is initiated means that the behaviour at the interface between liquid crystal and sheath solution is critical to the success of coaxial spinning. If the LC and polymer solvent are too miscible, the coaxial structure is lost and the LC acts as an internal coagulation bath for the polymer solution; if they are completely immiscible, the interfacial tension is too high. To achieve the ideal situation of a small miscibility gap in which nematic and isotropic liquids with slightly different compositions coexist with small interfacial tension between them, the chemical design of the mesogens used must be carefully considered in relation to the polymer – solvent combination used for the sheath. The peculiar nature of 5CB (and other *n*(O)CBs) may be both an asset and a problem in this context, and most likely it is best used as a component in a mixture that also includes mesogens of different designs.

## 4. Bio and gas sensing with 5CB

The VOC sensing experiments described above were very much inspired by the pioneering work in Nick Abbott’s group in using 5CB for detecting the presence of a variety of relevant analytes [37], from biomolecules [38–45] to liquid chemicals [46–48] and VOCs [49–52]. Several other groups have explored sensing with liquid crystals as well, often inspired by these seminal works [53–70]. While many of the principles behind LC-based sensing would probably work with other mesogens generating the desired phase, especially those that rely on amphiphilic molecules like lipids inducing an alignment transition from tangential to normal, one notes that 5CB or other *n*(O)CB mesogens, sometimes as components of mixtures such as E7, are almost omnipresent as the liquid crystal used in the sensing experiments. One may thus ask to what extent these works are ‘sensing with liquid crystals’ and how many are ‘sensing with *n*(O)CB’.

One subcategory that is definitely restricted to the *n*(O)CB architecture is the elegant gas sensing work in which the Abbott group used 5CB in contact with carefully selected substrates such as aluminium perchlorate. This is motivated by the fact that the cyano group forms a complex on such substrates, thus effectively creating an aligning surface of a monolayer of substrate-adsorbed 5CB which forces the bulk 5CB to align normal to the substrate. The beauty of this principle is that the targeted gases, in particular organophosphate nerve agents like sarin or their safer model compounds such as dimethyl methylphosphonate, form a *stronger* complex to the substrate than 5CB, hence exposure to the target gas releases the surface-adsorbed 5CB monolayer, changing the alignment at that substrate from normal to tangential. This leads to a clearly recognisable change in the polarising microscopy texture which then indicates to the user the presence of the target analyte.

Importantly, since this is a surface- rather than a bulk-driven change, very low threshold analyte concentrations can be expected, rendering these sensors particularly sensitive. The nematic-to-isotropic transition driving the macroscopic response in our LC core fibre mat VOC sensors described above is a bulk transition, thus exhibiting a greater threshold concentration, even though the extended surface-to-volume ratio of the fibre form factor helps. A particularly interesting way forwards may be to combine the two principles: spinning fibres with a sheath that induces a similar mesogen-specific binding to the aluminium perchlorate substrate. This would maximise the surface-to-volume ratio and place the responsive substrate on the outside, thus minimising the analyte diffusion time before the LC can respond, while taking advantage of the surface-driven sensing mechanism.

The Abbott team also found that the emulsions of 5CB droplets in water are exceptionally sensitive detectors of bacterial endotoxins, in this case responding to the Lipid A moiety that is common to all endotoxins [43]. It turns out that minute concentrations of Lipid A interacting with the 5CB droplets change the director field from a dipolar to a radial one, giving rise to a textural change that reveals the presence of endotoxin. While the small scale of the droplets requires a good-quality optical microscope to reveal the change, this is a very interesting sensing principle that is worth exploring further.

It would be interesting to expand liquid crystal-based sensing studies by a systematic variation of mesogenic structure, in particular exploring non-*n*(O)CB mesogens. This could then show where LC-based gas sensing is a reasonably generic concept, and where it is specific to the ‘fruit fly’ of LCs. It could well be that several examples of sensing with liquid crystals take advantage of the peculiar behaviour of *n*(O)CBs, without the exact mechanism being elucidated yet.

## 5. Dispersing nanoparticles in 5CB, 8CB or *n*CB mixtures

In the first years of the new century, the idea of using liquid crystals for simultaneously dispersing and aligning anisotropic nanoparticles, such as carbon nanotubes (CNTs) or graphene, motivated a new interdisciplinary research field for liquid crystal scientists. Among the pioneers of this development were Ingo Dierking and Giusy Scalia, who presented the first proof of principle [71,72] and several follow-up studies [73–79]. As usual when exploring a new idea involving liquid crystals, the question of which compounds to work with must be dealt with right away. In contrast to the cases of microfluidic shell production and fibre spinning, the LC is here not subject to flow, but there is still a need to work with large volumes. In this case, the main reason is that the relevant nanoparticle concentrations are so exceedingly small that LC volumes on the order of a mL are required to obtain the desired concentration, considering the minimum masses of nanoparticles that can be weighed out with any accuracy. Therefore, unsurprisingly, 5CB, E7 and other *n*CBs were the first mesogens to be explored, as they are available in large quantities at acceptable cost. In this case, the low clearing point of 5CB is an issue, since the dispersion of the nanoparticles typically requires ultrasonication, and this heats the sample quite significantly, such that 5CB may turn isotropic in the process. Because particles that are well dispersed in an isotropic liquid may get aggregated as a result of a transition to nematic, if the particles raise the energy of the nematic phase due to topological defects and elastic distortions [80,81], such a phase transition should generally be avoided. Thus, E7 or other LCs with high clearing points may be better choices simply from a phase sequence point of view.

The early experiments were highly encouraging, as the alignment of CNTs by the LC was confirmed and as the dispersibility was on par with many other organic solvents. Even though long-term stability cannot be ensured, except perhaps for vanishingly small particle concentrations, quite good dispersions of CNTs in E7 (and even 5CB, if sonication is done using pulse mode and with the sample immersed in an ice bath to avoid excessive heating) were found to be stable over hours to days. Scalia demonstrated using Raman spectroscopy on 5CB-dispersed CNTs that the interactions between the cyanobiphenyl unit and the aromatic CNT were strong and that the CNT peaks exhibit the same orientational order as the 5CB [79,82], hence the serendipitous choice to work with *n*CBs again turned out to be a quite good one (albeit not the best, as I will come back to in a moment). I was fortunate to collaborate with the Scalia group on LC-CNT dispersions for some time, my contributions being to focus on lyotropic LC hosts as well as on the relevance of the chemical mesogen design for the dispersability.

In the latter vein, we conducted a systematic study where we dispersed the same concentration of the same type of single-wall CNTs, under the same conditions, with no less than nine LC hosts, two of which were mixtures (E7 and RO-TN-403/015S, respectively) [83]. This led us to conclude that E7 is among the very best hosts for CNT dispersion, significantly better than 5CB, and we argued that this is a result of the favourable enthalpic interactions between LC and host (which E7 shares with 5CB) as well as favourable entropic interactions, where the mixture distinguishes itself from any single-component LC. The latter is an effect of the former, as the favourable enthalpic interactions lead to strong adsorption of mesogens onto the CNT surface, in an ordered fashion that may approach a 2D crystal arrangement for a single-component LC, yielding a significant entropy reduction given the large interfacial area in the case of well-dispersed CNTs. The reason that a multi-component mixture like E7 is a better choice from this perspective is that the different components all adsorb onto the CNT, preventing the 2D crystal formation and thus reducing the entropy loss.

Interestingly, one single-component LC faired particularly well in this study, and this was the classic (even older than 5CB) mesogen MBBA (*N*-(4-methoxybenzylidene)-4-butylaniline), which is not a cyanobiphenyl. Instead, it has a polar C=N bond at the centre, between the two phenyl rings, which we speculated may have been a reason for the excellent performance of MBBA as host for CNT dispersion. Perhaps, the steric hindrance of the hydrogens extending from the two phenyl rings in biphenyls, yielding a staggered rather than co-planar configuration of the *n*(O)CB core, is another reason for the better performance of MBBA, where the two phenyl rings are sufficiently separated to allow co-planar configurations, which should thus interact even stronger with the CNT surface.

An interesting side effect relating to *n*(O)CB mesogens that was triggered by our work in this field was the discovery that they can dramatically impact the phase diagram of other classes of mesogens, in this case a smectogen developing ferroelectric SmC* and antiferroelectric SmC a∗ phases [84]. We wanted to study the impact of CNT doping on these phases, but the minute quantities of smectogen available made it impossible to disperse the CNTs in this. To circumvent this problem, we therefore dispersed the CNTs in RO-TN-403/015S and then mixed this with the smectogen, which still developed the SmC* and SmC a∗ phases even with 20% (by mass) of the strictly nematic RO-TN-403/015S mixture added. To our great surprise, we found that the mixture also develops the complex ‘subphases’ SmC β∗ and SmC γ∗, intermediate between SmC ∗ and SmC a∗ [85], over broad temperature ranges. While we have encountered mixing-induced generation of these delicate subphases before by strategic combinations of smectogens that generate only SmC* and SmC a∗ phases, respectively [86], the idea that an *n*(O)CB-based nematic mixture would have the same impact is highly counterintuitive. The impact of *n*(O)CBs on differently designed mesogens generating entirely different phase sequences may be a very interesting area to explore further in the future. To the best of my knowledge, this has not been much investigated, but 5CB is now frequently being mixed with non-*n*(O)CB mesogens that also form a nematic phase, specifically reactive mesogens. I will end this perspective by discussing the modern use of *n*(O)CBs as a ‘solvent’ (which is quite different from the much earlier use of using liquid crystals as aligning solvents for NMR spectroscopy [87]).

## 6. Using 5CB and other *n*(O)CBs as solvent

The last decade has seen a surge in interest in liquid crystal networks (LCNs), soft rubbery elastomers (LCEs) as well as thin glassy films, most of them nematic although also cholesteric, and less frequently smectic, LCNs are also investigated. This development was largely driven by the 2015 LCE ‘click’ chemistry revolution introducing thiol-acrylate [88] and amine-acrylate [89] reactions to form the networks from commercially available monomeric reactive mesogens (‘RMs’), most often the diacrylates RM82 and RM257. While these mesogens are available in kg quantities at moderate prices, they do not have the convenient temperature range of 5CB, melting at 86°C (RM82) and 64°C (RM257), respectively. One must thus dilute or heat these mesogens for preparing the precursors. A not uncommon approach to circumvent this problem is to mix the reactive mesogens (and their reaction partners) with 5CB or another convenient *n*(O)CB or a mixture based on such mesogens. While they are not reactive and thus do not become a part of the final polymer, they serve as convenient ‘anisotropic solvents’ that reduce the melting point without removing liquid crystallinity. This yields precursor mixtures that are nematic (or cholesteric in the presence of chiral dopants), with convenient viscosity, at room temperature, and thus suitable for easy processing.

The team of Nathalie Katsonis demonstrated several examples of LCNs that exhibit spectacular light-induced shape morphing, made from mixtures of azobenzene diacrylates dissolved in E7 [90,91]. These mixtures can be conveniently filled into standard liquid crystal cells with rubbed polymer alignment layers, in their case imposing a twisted configuration that was then made permanent by polymerisation of the reactive components. By then opening the cell and cutting strips from the polymer-E7 film along different directions, LCNs that bend or curl in different ways in response to UV light exposure were obtained.

Shu Yang used 5CB together with a chiral dopant as cholesteric solvent for a reactive oligomer formed by RMs and an amine, again with the beneficial consequence that the mixture could easily be filled into a cell for ensuring good alignment of the mixture [92]. After polymerising and crosslinking the oligomer into a network and opening the cell, the unreacted 5CB and chiral dopant were removed by washing in ethanol. The resulting cholesteric liquid crystal elastomer (CLCE) films, which exhibited intense structural colouration, were then sandwiched on a mechanically robust transparent polydimethylsiloxane (PDMS) film which was then used to seal an airtight chamber in which air could be pumped at controlled pressure. This allowed them to strain the CLCE pneumatically to vary the reflection colour dynamically at will.

Devesh Mistry and co-workers in Helen Gleeson’s group used 6OCB as solvent to make nematic LCEs based on monoacrylate-terminated cyanobiphenyls [93,94]. In their case too, the non-reactive *n*OCB solvent was removed after polymerisation. The spectacular aspect of the LCEs produced in this way is that they exhibit auxetic behaviour, i.e. they show a region with negative Poisson’s ratio, where stretching leads to *expansion* rather than contraction along the perpendicular direction, as for conventional rubbers. Moreover, at the border between conventional and auxetic behaviour the system goes through a state of negative order parameter, which is another very exotic behaviour of these LCEs.

A slightly different way of using *n*CB as solvent in polymerised LCNs was presented by the Eindhoven groups around Dirk Broer, Albert Schenning and Danqing Liu [95,96]. The non-reactive character here had a very special role, namely that of a swelling agent which the LCN expels upon actuation, yielding a polymer that can ‘secrete’ *n*CB. This is an interesting proof of concept for on-demand liquid release driven by the phase transition of the LCN.

**Figure 5.**
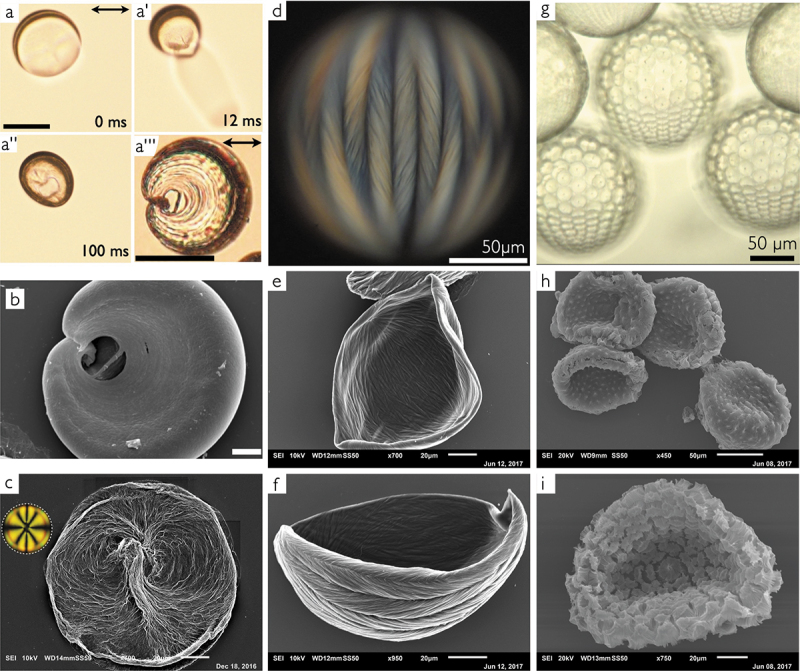
(Colour online) Shells of 5CB (a–b) and 8CB (c–i) that were polymer-stabilized by photopolymerizing the reactive mesogen RM257 dissolved in the LC. Panels (a–a  ′′) show still frames from a video (time indications at bottom right) in which a polymer-stabilized 5CB shell ruptures as a result of the inward-directed osmotic water flow taking place when the shell is immersed in pure water, due to the osmotic pressure caused by the internal phase containing 1 wt.% PVA. The hole forms in the defect-rich thinnest region. Scale bar: 100 *μ*m. The shell is washed in isopropanol to remove the 5CB and then dried, after which it is imaged from the side in optical microscopy (a  ′′′) and SEM (b). Scale bars: 500 *μ*m. (c) polymer-stabilizing a fully tangential-aligned nematic 8CB shell followed by rinsing, freeze-drying and 8CB removal (see main text) yields a network that clearly templates the director field of the nematic shell. The inset shows a polarizing microscopy image of a shell of the same type prior to polymerization, aligned in the same way as the shell in the SEM image. (d) a fully tangential-aligned 8CB shell in the SmA phase with 10 wt.% RM257 that has been photopolymerized into a network templated by the characteristic frustrated smectic order due to the confinement in the shell, imaged by polarizing microscopy. (e–f) shells produced in the same way and rinsed, freeze-dried and with the 8CB removed in the same procedure as before, and then imaged by SEM. (g) if one interface is normal-aligned, as ensured by stabilizing with an ionic surfactant on that side, the smectic 8CB shells develop a hybrid alignment in which a regular array of focal conic defects form. Here such shells are imaged from the side by optical microscopy (no analyzer). (h–i) the corresponding SEM images after the same rinsing, freeze-drying and 8CB removal process as before. All images were taken by JungHyun Noh when she was a member of our group.

To come full circle, let me end with some spectacular images taken by my former PhD student JungHyun Noh, demonstrating how polymerisation of a reactive mesogen dissolved in 5CB or 8CB can capture the structure formed by this extraordinary ‘solvent’ when moulded into a shell subject to varying boundary conditions. In this case, the polymer network polymer-stabilises the shell, being templated by the particular way in which the liquid crystal order has adapted to the shell confinement. In a tangentially aligned nematic 5CB shell the polymer network follows the director field with high fidelity as it curves around the shell, also reproducing the topological defects near the thinnest point. After polymerisation, JungHyun first placed the shell in pure water, causing an osmotic pressure gradient driving water into the shell due to the PVA dissolved in the internal aqueous phase. Because of the polymer network, the shell is no longer free to expand as when it is a fully monomeric liquid crystal, and when the internal pressure is sufficiently strong the shell ruptures near the defect-rich thin part, and the pressurised internal phase is expelled as seen in Figure 5a. Because of the particular way the director field is deformed around the defects, the hole acquires a ‘figure of 8’-like shape, seen from the top in a  ′′ and from the side in a  ′′′ and in b, the latter imaged by Scanning Electron Microscopy (SEM).

JungHyun also prepared 8CB shells with different stabilisers and then polymer-stabilised them in the nematic and the smectic state, respectively, see Figure 5(b–i). In this case, she did not want to break the shells, but she wished to image the polymer network by SEM with the shell intact. To this end, she washed the shell after photopolymerisation of the dissolved RM257 very briefly, just to remove excess PVA on the outside, and then quench-freezed the shells by dropping them into liquid nitrogen. This allowed her to freeze-dry the shells to remove all water without breaking them, after which she rinsed the shells in isopropanol to remove the unpolymerised 8CB prior to SEM imaging. While the shells did not break in the process, they typically collapsed, hence they obtained a ‘bowl-like’ shape in the final images. Panel (c) shows the curving polymer network templated by a shell that had been heated to the nematic state prior to photopolymerisation of the RM257, with all defects collecting near the thinnest part. It is easy to recognise the characteristic director field around the defects, as sketched in Figure 2(b) ′ (note that the shell in Figure 5(c) is rotated by 90° compared to Figure 2(b) ′, as illustrated by the inset polarising microscopy image in Figure 5(c)).

If the photopolymerisation is instead done in the smectic phase of a fully tangential-aligned 8CB shell, the polymer network adopts a ‘braided basket’-like chevron-striped texture, templated by the lunes that 8CB forms in the SmA phase in response to the frustration caused when the smectic layers must form in a tangential-aligned shell with radially changing surface area [16,17]. The polarising microscopy image of a shell with 10% RM257 after photopolymerisation is shown in Figure 5(d) and SEM images of two shells polymerised in this way, followed by the brief water rinsing, freeze-drying and 8CB dissolution by isopropanol, are shown in panels (e – f). If instead one interface is normal-aligned while the other is tangentially aligned, as achieved by using a surfactant as stabiliser on one side and PVA on the other, then the liquid crystal in the shell develops a regular array of focal conic defects in the SmA phase [18,97], see the optical microscopy image in Figure 5(g). After the shells are rinsed and freeze-dried and the 8CB is removed by isopropanol washing, SEM imaging reveals that the polymer network has now been templated by the focal conic defects into arrays of softly curved cones, see panels (h – i). These experiments show that 8CB (and other *n*(O)CBs) can be used to template highly complex structures when moulded into shell shape and then combined with reactive mesogens, an opportunity that we so far have only started to explore [98].

## 7. Concluding remarks

While this has largely been a summary of my personal story of 5CB and its homologues, I hope that the examples provided illustrate what a spectacular family of mesogens that they constitute, thereby providing a worthy contribution to this anniversary issue. I have tried to emphasise how 5CB, 8CB and the other ‘standards’ take on many roles, from being the standard choice when an LC is needed thanks to its convenient properties, ready availability and reasonable price, to providing very specific interactions with water or metal salt surfaces that enable results that are simply not achievable with other mesogens. Despite being middle-aged by now, there seems to be no sign that 5CB is about to retire. At least among academic researchers it is very much en vogue, and surely it will remain so for long. An interesting aspect that I did not go into in this article is that 5CB is currently rarely used in actual commercial devices. The future will tell which of the many new ideas for liquid crystal applications that have been presented over recent years will make it to the market, and whether 5CB will then become a device material again or not.

## Note

1. When applied to LC shells, PVA is truly just a stabiliser, not an alignment agent, as seen by its ability to allow tangential as well as normal alignment of an *n*CB-based LC stabilised by it [4,99].

## References

[1] Sluckin TJ, Dunmur DA, Stegemeyer H. Crystals that flow: classic papers from the history of liquid crystals. London: Taylor and Francis; 2004.

[2] Dunmur DA, Toriyama K. Light scattering and dielectric studies of molecular association in mesogenic solutions. Liq Cryst. 1986;1(2):169180. doi: 10.1080/02678298608086503

[3] Wilson MR, Dunmur DA. Molecular mechanics modelling of structure/property relationships in liquid crystals. Liq Cryst. 1989;5(3):987999. doi: 10.1080/02678298908026403

[4] Noh J, Wang Y, Liang HL, et al. Dynamic tuning of the director field in liquid crystal shells using block copolymers. Phys Rev Res. 2020;2(3):033160. doi: 10.1103/PhysRevResearch.2.033160

[5] Sharma A, Kizhakidathazhath R, Lagerwall J. Impact of mesogenic aromaticity and cyano termination on the alignment and stability of liquid crystal shells. Soft Matter. 2023 Apr;19(14):26372645. doi: 10.1039/D3SM00041A 36960755

[6] Blanc C, Durey G, Kamien RD, et al. Helfrich-hurault elastic instabilities driven by geometrical frustration. Rev Mod Phys. 2023;95(1). doi: 10.1103/RevModPhys.95.015004

[7] Agha H, Geng Y, Ma X, et al. Unclonable human-invisible machine vision markers leveraging the omnidirectional chiral Bragg diffraction of cholesteric spherical reflectors. Light Sci Appl. 2022;11(11):309. doi: 10.1038/s41377-022-01002-4 36284089 PMC9592545

[8] Schwartz M, Lenzini G, Geng Y, et al. Cholesteric liquid crystal shells as enabling material for information-rich design and architecture. Adv Mater. 2018 May;30(30):1707382. doi: 10.1002/adma.201707382 29756303

[9] Urbanski M, Reyes CG, Noh J, et al. Liquid crystals in micron-scale droplets, shells and fibers. J Phys. 2017;29(13):133003. doi: 10.1088/1361-648X/aa5706 28199222

[10] Lopez-Leon T, Fernandez-Nieves A. Drops and shells of liquid crystal. Colloid Polym Sci. 2011;289(4):345359. doi: 10.1007/s00396-010-2367-7

[11] Nelson DR. Toward a tetravalent chemistry of colloids. Nano Lett. 2002;2(10):11251129. doi: 10.1021/nl0202096

[12] Fernandez-Nieves A, Vitelli V, Utada A, et al. Novel defect structures in nematic liquid crystal shells. Phys Rev Lett. 2007;99(15):157801. doi: 10.1103/PhysRevLett.99.157801 17995213

[13] Lopez-Leon T, Koning V, Devaiah KBS, et al. Frustrated nematic order in spherical geometries. Nat Phys. 2011;7:391394. doi: 10.1038/nphys1920

[14] He K, Campo-Corts F, Goral M, et al. Micron-sized double emulsions and nematic shells generated via tip streaming. Phys Rev Fluids. 2019;4(12):124201. doi: 10.1103/PhysRevFluids.4.124201

[15] Liang HL, Enz E, Scalia G, et al. Liquid crystals in novel geometries prepared by microfluidics and electrospinning. Mol Cryst Liq Cryst. 2011;549:6977. doi: 10.1080/15421406.2011.581140

[16] Liang HL, Schymura S, Rudquist P, et al. Nematic-smectic transition under confinement in liquid crystalline colloidal shells. Phys Rev Lett. 2011;106(24):247801. doi: 10.1103/PhysRevLett.106.247801 21770600

[17] Lopez-Leon T, Fernandez-Nieves A, Nobili M, et al. Nematic-smectic transition in spherical shells. Phys Rev Lett. 2011;106(24):247802. doi: 10.1103/PhysRevLett.106.247802 21770601

[18] Liang H, Noh J, Zentel R, et al. Tuning the defect configurations in nematic and smectic liquid crystalline shells. Philos Trans A. 2013 Apr;371(1988):20120258. doi: 10.1098/rsta.2012.0258 23459961

[19] Thomsen DL III, Keller P, Naciri J, et al. Liquid crystal elastomers with mechanical properties of a muscle. Macromolecules. 2001;34(17):58685875. doi: 10.1021/ma001639q

[20] Fleischmann EK, Liang HL, Kapernaum N, et al. One-piece micropumps from liquid crystalline core-shell particles. Nat Commun. 2012;3(1):1178. doi: 10.1038/ncomms2193 23132028

[21] Sharma A, Stoffel AM, Lagerwall JP. Liquid crystal elastomer shells with topological defect-defined actuation: complex shape morphing, opening/closing, and unidirectional rotation. J Appl Phys. 2021;129(17):174701. doi: 10.1063/5.0044920

[22] Brake J, Abbott N. An experimental system for imaging the reversible adsorption of amphiphiles at aqueous-liquid crystal interfaces. Langmuir. 2002;18(16):61016109. doi: 10.1021/la011746t

[23] Enz E, Baumeister U, Lagerwall J. Coaxial electrospinning of liquid crystal-containing poly(vinyl pyrrolidone) microfibers. Beilstein J Org Chem. 2009;5(58). doi: 10.3762/bjoc.5.58 PMC2839530 20300504

[24] Schelski K, Reyes CG, Pschyklenk L, et al. Quantitative volatile organic compound sensing with liquid crystal core fibers. Cell Rep Physical Sci. 2021 Nov;2(12):100661. doi: 10.1016/j.xcrp.2021.100661 PMC8724680 35028624

[25] Gerber P. Measurement of the rotational viscosity of nematic liquid-crystals. Appl Phys A. 1981;26(3):139–142. doi: 10.1007/BF00614747

[26] Lagerwall JPF, McCann JT, Formo E, et al. Coaxial electrospinning of microfibres with liquid crystal in the core. Chem Commun. 2008;42:54205422. doi: 10.1039/b810450f 18985230

[27] Enz E, Lagerwall J. Electrospun microfibres with temperature sensitive iridescence from encapsulated cholesteric liquid crystal. J Mater Chem. 2010;20(33):68666872. doi: 10.1039/c0jm01223h

[28] Scalia G, Enz E, Cal O, et al. Morphology and core continuity of liquid-crystal-functionalized, coaxially electrospun fiber mats tuned via the polymer sheath solution. Macromol Mater Eng. 2013;298(5):583589. doi: 10.1002/mame.201200361

[29] Buyuktanir E, Frey M, West J. Self-assembled, optically responsive nematic liquid crystal/polymer core-shell fibers: Formation and characterization. Polymer. 2010;51(21):48234830. doi: 10.1016/j.polymer.2010.08.011

[30] Kim DK, Hwang M, Lagerwall JPF. Liquid crystal-functionalization of electrospun polymer fibers. J Polym Sci B. 2013;51(11):855867. doi: 10.1002/polb.23285

[31] Reyes CG, Sharma A, Lagerwall JP. Non-electronic gas sensors from electrospun mats of liquid crystal core fibers for detecting volatile organic compounds at room temperature. Liq Cryst. 2016;43(1315):19862001. doi: 10.1080/02678292.2016.1212287

[32] Reyes CG, Lagerwall JP. Advancing flexible volatile compound sensors using liquid crystals encapsulated in polymer fibers. In: Chien L-C, editor. Emerging liquid crystal technologies XIII. Vol. 10555. International Society for Optics and Photonics: 2018. p. 1055500.

[33] Wang J, Jkli A, West JL. Liquid crystal/polymer fiber mats as sensitive chemical sensors. J Mol Liq. 2018;267:490495. doi: 10.1016/j.molliq.2018.01.051

[34] Reyes C, Lagerwall J. Disruption of electrospinning due to water condensation into the Taylor cone. ACS Appl Mater Interfaces. 2020 Jun;12(23):2656626576. doi: 10.1021/acsami.0c03338 PMC7302509 32420728

[35] Reyes C, Baller J, Araki T, et al. Isotropic-isotropic phase separation and spinodal decomposition in liquid crystal-solvent mixtures. Soft Matter. 2019 Jun;15(30):60446054. doi: 10.1039/C9SM00921C 31225565

[36] Vats S, Anyfantakis M, Honaker L, et al. Stable electrospinning of core-functionalized coaxial fibers enabled by the minimum-energy interface given by partial core-sheath miscibility. Langmuir. 2021;37(45):13265–13277. doi: 10.1021/acs.langmuir.1c01824 34735163 PMC8600680

[37] Carlton RJ, Hunter JT, Miller DS, et al. Chemical and biological sensing using liquid crystals. Liq Cryst Rev. 2013;1(1):2951. doi: 10.1080/21680396.2013.769310 PMC4005293 24795857

[38] Brake J, Daschner M, Luk Y, et al. Biomolecular interactions at phospholipid-decorated surfaces of liquid crystals. Science. 2003;302(5653):20942097. doi: 10.1126/science.1091749 14684814

[39] Luk Y, Abbott N, Bertics P, et al. Using liquid crystals and nanostructured surfaces to detect regulatory proteins involved in cell signaling pathways. Biochemistry. 2003;42(28):86388638.

[40] Jang C, Cheng L, Olsen C, et al. Anchoring of nematic liquid crystals on viruses with different envelope structures. Nano Lett. 2006;6(5):10531058. doi: 10.1021/nl060625g 16683850

[41] Park J, Abbott N. Ordering transitions in thermotropic liquid crystals induced by the interfacial assembly and enzymatic processing of oligopeptide amphiphiles. Adv Mater. 2008;20(6):1185. doi: 10.1002/adma.200702012

[42] Sivakumar S, Wark K, Gupta J, et al. Liquid crystal emulsions as the basis of biological sensors for the optical detection of bacteria and viruses. Adv Funct Mater. 2009;19(14):22602265. doi: 10.1002/adfm.200900399

[43] Lin IH, Miller D, Bertics P, et al. Endotoxin-induced structural transformations in liquid crystalline droplets. Science. 2011;332(6035):12971300. doi: 10.1126/science.1195639 PMC3448959 21596951

[44] Jiang S, Noh J, Park C, et al. Using machine learning and liquid crystal droplets to identify and quantify endotoxins from different bacterial species. Analyst. 2021 Feb;146(4):12241233. doi: 10.1039/D0AN02220A 33393547

[45] Kim Y, Wang X, Mondkar P, et al. Self-reporting and self-regulating liquid crystals. Nature. 2018 May;557(7706):539544. doi: 10.1038/s41586-018-0098-y 29743674

[46] Shah R, Abbott N. Using liquid crystals to image reactants and products of acid-base reactions on surfaces with micrometer resolution. J Am Chem Soc. 1999;121(49):1130011310. doi: 10.1021/ja9844837

[47] Shah R, Abbott N. Principles for measurement of chemical exposure based on recognition-driven anchoring transitions in liquid crystals. Science. 2001;293(5533):12961299. doi: 10.1126/science.1062293 11509724

[48] Manna U, Zayas-Gonzalez Y, Carlton R, et al. Liquid crystal chemical sensors that cells can wear. Angew Chem Int Ed Engl. 2013 Dec;52(52):140115. doi: 10.1002/anie.201306630 24288229

[49] Yang KL, Cadwell K, Abbott NL. Mechanistic study of the anchoring behavior of liquid crystals supported on metal salts and their orientational responses to dimethyl methylphosphonate. J Phys Chem B. 2004;108(52):2018020186. doi: 10.1021/jp0470391

[50] Cadwell KD, Lockwood NA, Nellis BA, et al. Detection of organophosphorous nerve agents using liquid crystals supported on chemically functionalized surfaces. Sens Actuat B. 2007;128(1):9198. doi: 10.1016/j.snb.2007.05.044

[51] Hunter JT, Pal SK, Abbott NL. Adsorbate-induced ordering transitions of nematic liquid crystals on surfaces decorated with aluminum perchlorate salts. ACS Appl Mater Interfaces. 2010;2(7):18571865. doi: 10.1021/am100165a

[52] Hunter J, Abbott N. Dynamics of the chemo-optical response of supported films of nematic liquid crystals. Sens Actuat B-Chem. 2013;183:7180. doi: 10.1016/j.snb.2013.03.094

[53] Niu X, Zhong Y, Chen R, et al. Highly sensitive and selective liquid crystal optical sensor for detection of ammonia. Opt Express. 2017;25(12):13549–13556. doi: 10.1364/OE.25.013549 28788898

[54] Kim H, Jang C. Liquid crystal-based capillary sensory platform for the detection of bile acids. Chem Phys Lipids. 2017;204:10–14. doi: 10.1016/j.chemphyslip.2017.02.003 28209390

[55] Kim HJ, Jang CH. Micro-capillary sensor for imaging trypsin activity using confined nematic liquid crystals. J Mol Liq. 2016;222:596–600. doi: 10.1016/j.molliq.2016.07.099

[56] Ramou E, Roque ACA. Textural landscapes of voc-sensitive chiral liquid crystal-based materials. Appl Phys Rev. 2023;10(1):011411. doi: 10.1063/5.0136551

[57] Paterson DA, Du X, Bao P, et al. Chiral nematic liquid crystal droplets as a basis for sensor systems. Mol Syst Des Eng. 2022;7:607–621. doi: 10.1039/D1ME00189B 36876150 PMC9972830

[58] Esteves C, Ramou E, Porteira ARP, et al. Seeing the unseen: the role of liquid crystals in gas sensing technologies. Adv Opt Mater. 2020;8(11):1902117. doi: 10.1002/adom.201902117 32612901 PMC7329384

[59] Bao P, Paterson D, Harrison P, et al. Lipid coated liquid crystal droplets for the on-chip detection of antimicrobial peptides. Lab Chip. 2019;19(6):1082–1089. doi: 10.1039/C8LC01291A 30785139 PMC6484679

[60] Popov P, Honaker LW, Kooijman EE, et al. A liquid crystal biosensor for specific detection of antigens. Sens Bio-Sens Res. 2016;8:31–35. doi: 10.1016/j.sbsr.2016.03.008

[61] Popov N, Honaker LW, Popova M, et al. Thermotropic liquid crystal-assisted chemical and biological sensors. Materials. 2017;11(1):20. doi: 10.3390/ma11010020 29295530 PMC5793518

[62] Hussain A, Semeano A, Palma S, et al. Tunable gas sensing gels by cooperative assembly. Adv Funct Mater. 2017;27(27):27. doi: 10.1002/adfm.201700803 PMC5524183 28747856

[63] Jang JH, Park SY. ph-responsive cholesteric liquid crystal double emulsion droplets prepared by microfluidics. Sens Actuat B. 2017;241:636–643. doi: 10.1016/j.snb.2016.10.118

[64] Patrick NS, Richard RH, Daniel SK. Liquid crystal reorientation induced by aptamer conformational changes. J Am Chem Soc. 2013;135(13):5183–5189. doi: 10.1021/ja400619k 23510322

[65] Alino VJ, Sim PH, Choy WT, et al. Detecting proteins in microfluidic channels decorated with liquid crystal sensing dots. Langmuir. 2012;28(50):17571–17577. doi: 10.1021/la303213h 23163482

[66] Aaron MC, Patrick NS, Daniel SK. Surfactant-DNA interactions at the liquid crystal-aqueous interface. Soft Matter. 2012;8(16):4335–4342. doi: 10.1039/c2sm07483d

[67] Stephanie MM, Daniel SK. Macroscopic liquid crystal response to isolated DNA helices. Langmuir. 2011;27(19):11767–11772. doi: 10.1021/la202640a 21894894

[68] Alino VJ, Pang J, Yang KL. Liquid crystal droplets as a hosting and sensing platform for developing immunoassays. Langmuir. 2011;27(19):11784–11789. doi: 10.1021/la2022215 21863867

[69] Andrew PD, Ignes-Mullol J, Vallve MA, et al. Liquid crystal anchoring transformations induced by phase transitions of a photoisomerizable surfactant at the nematic/aqueous interface. Soft Matter. 2009;5(11):2252–2260. doi: 10.1039/b821980j

[70] Price A, Schwartz D. DNA hybridization-induced reorientation of liquid crystal anchoring at the nematic liquid crystal/aqueous interface. J Am Chem Soc. 2008;130(26):8188–8194. doi: 10.1021/ja0774055 18528984

[71] Dierking I, Scalia G, Morales P. Liquid crystal-carbon nanotube dispersions. J Appl Phys. 2005;97:044309. doi: 10.1063/1.1850606

[72] Dierking I, Scalia G, Morales P, et al. Aligning and reorienting carbon nanotubes with nematic liquid crystals. Adv Mater. 2004;16(11):865–869. doi: 10.1002/adma.200306196

[73] Yakemseva M, Dierking I, Kapernaum N, et al. Dispersions of multi-wall carbon nanotubes in ferroelectric liquid crystals. Eur Phys J E. 2014;37(2). doi: 10.1140/epje/i2014-14007-4 24532223

[74] Schymura S, Scalia G. On the effect of carbon nanotubes on properties of liquid crystals. Philos Trans A. 2013;371(1988):20120261. doi: 10.1098/rsta.2012.0261 23459963

[75] Scalia G. Alignment of carbon nanotubes in thermotropic and lyotropic liquid crystals. Chemphyschem. 2010;11(2):333–340. doi: 10.1002/cphc.200900747 20013984

[76] Dierking I, Casson K, Hampson R. Reorientation dynamics of liquid crystal-nanotube dispersions. Jpn J Appl Phys. 2008;47(8):6390–6393. doi: 10.1143/JJAP.47.6390

[77] Scalia G, Lagerwall JPF, Schymura S, et al. Carbon nanotubes in liquid crystals as versatile functional materials. Phys Stat Sol (B). 2007;244(11):4212–4217. doi: 10.1002/pssb.200776205

[78] Dierking I, San S. Magnetically steered liquid crystal-nanotube switch. Appl Phys Lett. 2005;87(23):233507. doi: 10.1063/1.2140069

[79] Scalia G, Haluska M, Dettlaff-Weglikowska U, et al. Polarized Raman spectroscopy study of swcnt orientational order in an aligning liquid crystalline matrix. AIP Conf Proc. 2005;786:114–117.

[80] Petrov P, Terentjev EM. Formation of cellular solid in liquid crystal colloids. Langmuir. 2001;17(10):2942–2949. doi: 10.1021/la0016470

[81] Meeker SP, Poon WCK, Crain J, et al. Colloid-liquid-crystal composites: an unusual soft solid. Phys Rev E. 2000;61(6):R6083–R6086. doi: 10.1103/PhysRevE.61.R6083 11088356

[82] Scalia G, Lagerwall JPF, Haluska M, et al. Effect of phenyl rings in liquid crystal molecules on swcnts studied by Raman spectroscopy. Phys Stat Sol (B). 2006;243(13):3238–3241. doi: 10.1002/pssb.200669205

[83] Schymura S, Kühnast M, Lutz V, et al. Towards efficient dispersion of carbon nanotubes in thermotropic liquid crystals. Adv Funct Mater. 2010;20(19):3350–3357. doi: 10.1002/adfm.201000539

[84] Lagerwall JPF, Dabrowski R, Scalia G. Antiferroelectric liquid crystals with induced intermediate polar phases and the effects of doping with carbon nanotubes. J Non-Cryst Solids. 2007;353(47–51):4411–4417. doi: 10.1016/j.jnoncrysol.2007.01.094

[85] Lagerwall JPF, Giesselmann F. Current topics in smectic liquid crystal research. Chemphyschem. 2006;7(1):20–45. doi: 10.1002/cphc.200500472 16404767

[86] Lagerwall JPF, Heppke G, Giesselmann F. Frustration between syn- and anticlinicity in mixtures of chiral and non-chiral tilted smectic-c-type liquid crystals. Eur Phys J E. 2005;18(1):113–121. doi: 10.1140/epje/i2005-10035-5 16211333

[87] Suryaprakash N. Liquid crystals as solvents in NMR spectroscopy: current developments in structure determination. Curr Org Chem. 2000;4(1):85–103. doi: 10.2174/1385272003376373

[88] Yakacki CM, Saed M, Nair DP, et al. Tailorable and programmable liquid-crystalline elastomers using a two-stage thiol–acrylate reaction. RSC Adv. 2015;5(25):18997–19001. doi: 10.1039/C5RA01039J PMC4781659 26862925

[89] Ware TH, McConney ME, Wie JJ, et al. Actuating materials. voxelated liquid crystal elastomers. Science. 2015;347(6225):982–984. doi: 10.1126/science.1261019 25722408

[90] Aßhoff S, Lancia F, Iamsaard S, et al. High-power actuation from molecular photoswitches in enantiomerically paired soft springs. Angew Chem Int Ed. 2017;56(12):3261–3265. doi: 10.1002/anie.201611325 PMC5363340 28181400

[91] Iamsaard S, Aßhoff S, Matt B, et al. Conversion of light into macroscopic helical motion. Nat Chem. 2014;6(3):229–235. doi: 10.1038/nchem.1859 24557138

[92] Kim SU, Lee YJ, Liu J, et al. Broadband and pixelated camouflage in inflating chiral nematic liquid crystalline elastomers. Nat Mater. 2022;21(1):41–46. doi: 10.1038/s41563-021-01075-3 34489567

[93] Mistry D, Morgan PB, Clamp JH, et al. New insights into the nature of semi-soft elasticity and “mechanical-fréedericksz transitions” in liquid crystal elastomers. Soft Matter. 2018;14(8):1301–1310. doi: 10.1039/C7SM02107K 29368788

[94] Mistry D, Connell SD, Mickthwaite S, et al. Coincident molecular auxeticity and negative order parameter in a liquid crystal elastomer. Nat Commun. 2018;9(1):5095. doi: 10.1038/s41467-018-07587-y 30514842 PMC6279820

[95] Gelebart AH, Liu D, Mulder DJ, et al. Photoresponsive sponge-like coating for on-demand liquid release. Adv Funct Mater. 2018;28(10):1705942. doi: 10.1002/adfm.201705942

[96] Zhan Y, Zhou G, Lamers B, et al. Artificial organic skin wets its surface by field-induced liquid secretion. Matter. 2020;3(3):782–793. doi: 10.1016/j.matt.2020.05.015 32954253 PMC7487776

[97] Noh J, Lagerwall JP. Topological defect-guided regular stacking of focal conic domains in hybrid-aligned smectic liquid crystal shells. Crystals. 2021;11(8):913. doi: 10.3390/cryst11080913

[98] Noh J, Henx B, Lagerwall JP. Taming liquid crystal self-assembly: the multifaceted response of nematic and smectic shells to polymerization. Adv Mater. 2016;28(46):10170–10174. doi: 10.1002/adma.201603158 27689941

[99] Durey G, Ishii Y, Lopez-Leon T. Temperature-driven anchoring transitions at liquid crystal/water interfaces. Langmuir. 2020;36(32):93689376. doi: 10.1021/acs.langmuir.0c00985 32693599

